# The Viral Founder Effect and Economic-Driven Human Mobility Shaped the Distinct Epidemic Pattern of HIV-1 CRF01_AE in Northeast China

**DOI:** 10.3389/fmed.2021.769535

**Published:** 2021-12-03

**Authors:** Minghui An, Bin Zhao, Lin Wang, Zhenxing Chu, Junjie Xu, Haibo Ding, Xiaoxu Han, Hong Shang

**Affiliations:** ^1^NHC Key Laboratory of AIDS Immunology (China Medical University), National Clinical Research Center for Laboratory Medicine, The First Affiliated Hospital of China Medical University, Shenyang, China; ^2^Key Laboratory of AIDS Immunology, Chinese Academy of Medical Sciences, Shenyang, China; ^3^Key Laboratory of AIDS Immunology of Liaoning Province, Shenyang, China

**Keywords:** HIV-1, CRF01_AE, phylogeographic, phylodynamic, men who have sex with men, China

## Abstract

**Background:** In China, two distinct lineages shaped the epidemic of HIV-1 CRF01_AE among men who have sex with men (MSM), of which the uneven distributions were observed geographically. One lineage spread across China, while another dominated in Northeast China. Understanding the drivers of viral diffusion would provide guidelines for identifying the source and hotspots of HIV transmission among MSM to target interventions in China.

**Methods:** We collected the *pol* sequences between 2002–2017 to reconstruct the spatiotemporal history of CRF01_AE lineages in Shenyang, one economic center of Northeast China, using the Bayesian phylogeographic and phylodynamic approaches. Importantly, for the datasets with the high sample density, we did the down-sampling to avoid the sampling bias.

**Results:** Two lineages accounted for 97%, including 426 and 1516 sequences, and homosexuals and bisexuals were above 80%. One lineage appeared earlier 7 years than another (1993 vs. 2002) among homosexuals and bisexuals, whereas among heterosexuals, both lineages were observed firstly in 2002. 96% viral migrations within one lineage were from homosexuals toward bisexuals (49%) and male-heterosexuals (46%). Within another, except for homosexuals (72%), bisexuals (23%) served as the top second source, and female-heterosexuals (11%) were the third recipients following bisexuals (44%) and male-heterosexuals (39%). Although the basic reproduction number (R_0_) of two lineages were similar and both of the effective production number (R_e_) fell below 1 at the most recent sampling time, the starts of the R_e_ declining varied.

**Conclusions:** Our findings revealed that throughout the viral national spread chain, Shenyang is the source for the initial expanding of one lineage, where is only a sink of another, proving that the viral founder effect and regional human mobility contributed to the uneven distribution of two lineages, and emphasizing the important roles of the area where the virus originated and economy-driven migrants in HIV transmission.

## Introduction

According to a systematic review based on the global data collected from 1990-2015, HIV-1 CRF01_AE was the second-most prevalent circulating recombinant form (CRF) in the world, causing 5.3% of all HIV-1 infections (1). CRF01_AE originated in Central Africa in the early 1970s and then Thailand, served as a secondary distribution center, transmitted CRF01_AE to other countries, driving the global epidemic (2). Among all recombinant forms, the distribution of CRF01_AE included more than 50% of East Asia and nearly 90% of Southeast Asia (3).

In China, the national molecular epidemiological surveys revealed that CRF01_AE spread nationwide, becoming the second-most prevalent subtype, behind CRF07_BC, to date (4). Multiple independent CRF01_AE epidemics were likely introduced to China during the 1990s, which may have originated from Southeast Asia (5–7). The dominant risk groups associated with these CRF01_AE lineages were different, with two major lineages (L4 and L5) identified as more prevalent among men who have sex with men (MSM) and other lineages (L1-L3, L6-7) mainly circulating among heterosexuals and people who inject drugs (PWID) (5). Our multiple-center MSM survey found that although these two lineages have spread among MSM nationwide, their proportions differ across regions, with one observed at a higher density in Northeast China (7, 8).

Liaoning, located in Northeast China, featured 19,427 persons living with HIV, as of 2019, accounting for 2.4% of the total HIV population in China, and has a middle-level HV prevalence (4.4%) among MSM (9). MSM represent the predominant at-risk population, compared with heterosexuals, PWID and blood transfusion receivers. CRF01_AE has been found among MSM even since the early period of the HIV epidemic in Liaoning during the early 2000s (10). Currently, L4 and L5 combined represent > 70% of all HIV-1 infections in Liaoning, followed by CRF07_BC and subtype B. Moreover, L5 is much more than L 4 in Liaoning and other two neighboring northeastern provinces (7, 11, 12). Understanding which factors drive the uneven distribution for the two lineages and determining the epidemic patterns underlying viral spread are important for identifying transmission characteristics, which can guide public health responses and targeting interventions.

In this study, we analyzed a local HIV-1 drug-resistance database from 2002 to 2017 using Bayesian phylodynamic and phylogeographic approaches, to trace the spatiotemporal dynamics of CRF01_AE in Shenyang of Liaoning, one economic center in northeast China. We primarily focused on two issues: (1) the drivers of the uneven distribution of two HIV-1 CRF01_AE lineages (higher in Northeast China), and (2) the effectiveness of HIV interventions implemented among MSM in Shenyang.

## Methods

### Data Collection

All available HIV-1 CRF01_AE *pol* sequences (HXB2: 2253-3314 nt) surveyed between 2002–2017 were obtained from a Shenyang local HIV-1 drug-resistance database built by the First Hospital of China Medical University. All sequences were sampled from treatment naive patients at the time of diagnosis or at the first follow-up time, avoiding the convergent evolution induced by the drug selective pressure. Age, sex, risk factors and sampling date were collected from a local HIV demographic database. The use of viral sequences was approved and governed by the Medical Ethics Committee of the First Hospital of China Medical University.

### Identification of HIV-1 CRF01_AE Lineages

The sequences were aligned and edited using AliView v1.25. The best-fitting nucleotide substitution model was evaluated using the ModelFinder package in IQ-TREE v 1.6.12 (13). The maximum likelihood (ML) tree was reconstructed under the GTR+I+G4 model in IQ-Tree, and the branch support was tested using the approximate likelihood-ratio test (aLRT) with 1,000 replicates (14). The lineages were defined when aLRT > 95% and contained ≥2 local sequences. The reference sequences (7) used to distinguish the lineages and determine the tree topology were downloaded in the Los Alamos HIV database (http://www.hiv.lanl.gov).

### Time-Scaled Phylogenetic and Phylogeographic Inferences of CRF01_AE Lineages Among High-Risk Groups

The sequence datasets of major lineages were tested using TempEst (http://beast.community/tempest) to identify the temporal signal based on the positive correlation between genetic diversity and the sampling date. A local MSM high-risk cohort assembled in 2008, so a large quantity of sequences from homosexuals and bisexuals were obtained since 2008 (Table 1). Therefore, we performed a down-sampling procedure to avoid the over-sampling bias in homosexuals and bisexuals (Supplementary Table 1). The ancestral origins and evolutionary rates among different risk groups were estimated using the GTR+I+G4 model, a relaxed lognormal clock model and a Bayesian non-parametrized Skygrid model (15) in BEAST v1.10.4. The reference sequences from Africa and Thailand (16) were chosen to fix the topology of the maximum clade credibility (MCC) tree and to calibrate the time to most recent common ancestor (tMRCA). Viral migrations across risk groups were estimated using a discrete asymmetric substitution model by performing Bayesian stochastic search variable selection (BSSVS). The risk groups were classified as follows: homosexuals, bisexuals, heterosexuals, PWID, and blood transfusion receivers. The well-supported viral migration between risk groups was calculated by Bayes Factor (BF) values using SpreaD3 (17). The number of the transitions was computed using the Markov jumps method implemented in BEAST1.10.4. Markov chain Monte Carlo (MCMC) runs were performed until the effective sample size (ESS) >200, after excluding an initial 10% as burn-in, using Tracer 1.7.1. The fractions of viral migration events between sources and recipients were drawn using the Sankey plot in R version 4.0.2.

**Table 1:** Demographic characteristics of HIV-1 CRF01_AE in Shenyang.

	**All**	**Lineage 4**		**Lineage 5**		***p***
		***N***	**%**	***N***	**%**	
Sex						
Man	1,934	418	98.12%	1466	96.70%	0.129
Female	80	8	1.88%	50	3.30%	
Birth period						
Before 1960s	159	6	1.41%	145	9.56%	<0.001
1960s	333	38	8.92%	275	18.14%	
1970s	369	69	16.20%	280	18.47%	
1980s	779	215	50.47%	546	36.02%	
After 1990s	367	96	22.54%	265	17.48%	
Unknown	7	2	0.47%	5	0.33%	
Risk group^*^						
Homosexuals	1,422	326	76.53%	1077	71.04%	0.272
HET-male	182	34	7.98%	140	9.23%	
HET-female	76	8	1.88%	49	3.23%	
Bisexuals	308	57	13.38%	245	16.16%	
PWID	20	0	0.00%	2	0.13%	
Blood	6	1	0.23%	3	0.20%	
Household register						
Shenyang	1,866	402	94.37%	1403	92.55%	0.333
Other cities in Liaoning	126	19	4.46%	96	6.33%	
Other provinces	13	2	0.47%	11	0.73%	
Unknown	9	3	0.70%	6	0.40%	
Sampling date						
2002–2007	48	3	0.70%	35	2.31%	0.006
2008	94	8	1.88%	79	5.21%	
2009	126	17	3.99%	104	6.86%	
2010	119	26	6.10%	90	5.94%	
2011	114	22	5.16%	89	5.87%	
2012	167	35	8.22%	125	8.25%	
2013	195	52	12.21%	140	9.23%	
2014	319	77	18.08%	230	15.17%	
2015	301	64	15.02%	228	15.04%	
2016	284	72	16.90%	207	13.65%	
2017	248	50	11.74%	189	12.47%	
Total	2,014	426	100.00%	1516	100.00%	

* *HET, heterosexuals; PWID, person who inject drugs; Blood, blood transfusion receivers*.

### Phylodynamic Reconstruction of CRF01_AE Lineages

Birth-Death SIR (BDSIR) model was used to reconstruct the transmission dynamics of major lineages under the GTR+I+G4 model and a relaxed lognormal clock model in BESAT v2.6.3. SIR model divided the population into three compartments: susceptible, infectious and recovered (cured or death). Even if HIV infection can't be cured, it is believed that patients who achieve the virological suppression under the high active antiretroviral therapy (HAART) would not transmit the virus to others and they can be removed from the infectious pool as the recovered population. Liaoning initiated the HAART in 2003 for HIV infected patients in need and by the end 2012, the viral suppression rate was high to 95% (18). Nationally, the coverage of HAART among sexually active population in need was 61.7% before 2010 (19). Shenyang, as the capital city of Liaoning, has more standardized HIV treatment system, and moreover, the high risk cohort among MSM built since 2008 enhanced the related health care and intervention (20), both of which would contribute to the higher coverage and viral suppression rates of treatment in Shenyang. The SIR model is therefore suitable for the HIV status of Shenyang. Some key epidemiological parameters, such as the viral basic reproduction number (R_0_) and the effective reproduction number (R_e_) over time were estimated using Bayesian inference. The prior distributions of key epidemiological parameters were set as follows: a log-normal (8, 2) for the population size of susceptible individuals, with a 95% confidence interval (CI) between 59.1 and 150000; a log-normal (0, 1) for the becoming-non-infectious rate, with the median infectiousness duration of one year and a 95% confidence interval (CI) between 51 days and 7 years; an uniform (0, 50) for the origin of the epidemic, in view of the first reported HIV positive case of China in 1985; a log-normal (0, 1) for the reproduction number, with a 95% interval from 0.14 to 7.1; and a Beta (1, 10) for the sampling proportion, with a 95% interval between 0.3 to 31%. The analyses were run for at least 200 million until the until ESS >200 and the plots of BDSIR analysis were drawn using the phylodynamics script (https://github.com/BEAST2-Dev/phylodynamics) in R version 4.0.2.

### Statistical Analysis

The demographic information of individuals infected by the two main HIV-1 CRF01_AE lineages was compared using the Chi-square test. When the number was less than 5, Fisher's exact test was used. *P*-values < 0.05 denoted statistically significant differences.

### Sequence Accession Numbers

Most local sequences used in this study have been previously submitted to GenBank, and the accession numbers are listed in Supplementary Materials. Other local sequences have been submitted to GenBank, and the accession numbers are MW690216–MW690550.

## Results

### The Demographic Characteristics of CRF01_AE Lineages in Shenyang

A total of 2,672 CRF01_AE *pol* sequences were screened from a local HIV-1 drug-resistance database. After deleting duplicates and eliminating sequences without sex and risk group information, 2,014 sequences remained in Dataset A (Table 1). Half were born after 1980, and 99% were Liaoning residents. Male was the predominant sex, and homosexuals accounted for 70.6%, followed by bisexuals (15.3%), heterosexuals (12.8%), PWID (1%) and blood transfusion recipients (0.3%). The distributions of male and female were uneven among heterosexual population (9 vs. 4%).

**Figure 1:**
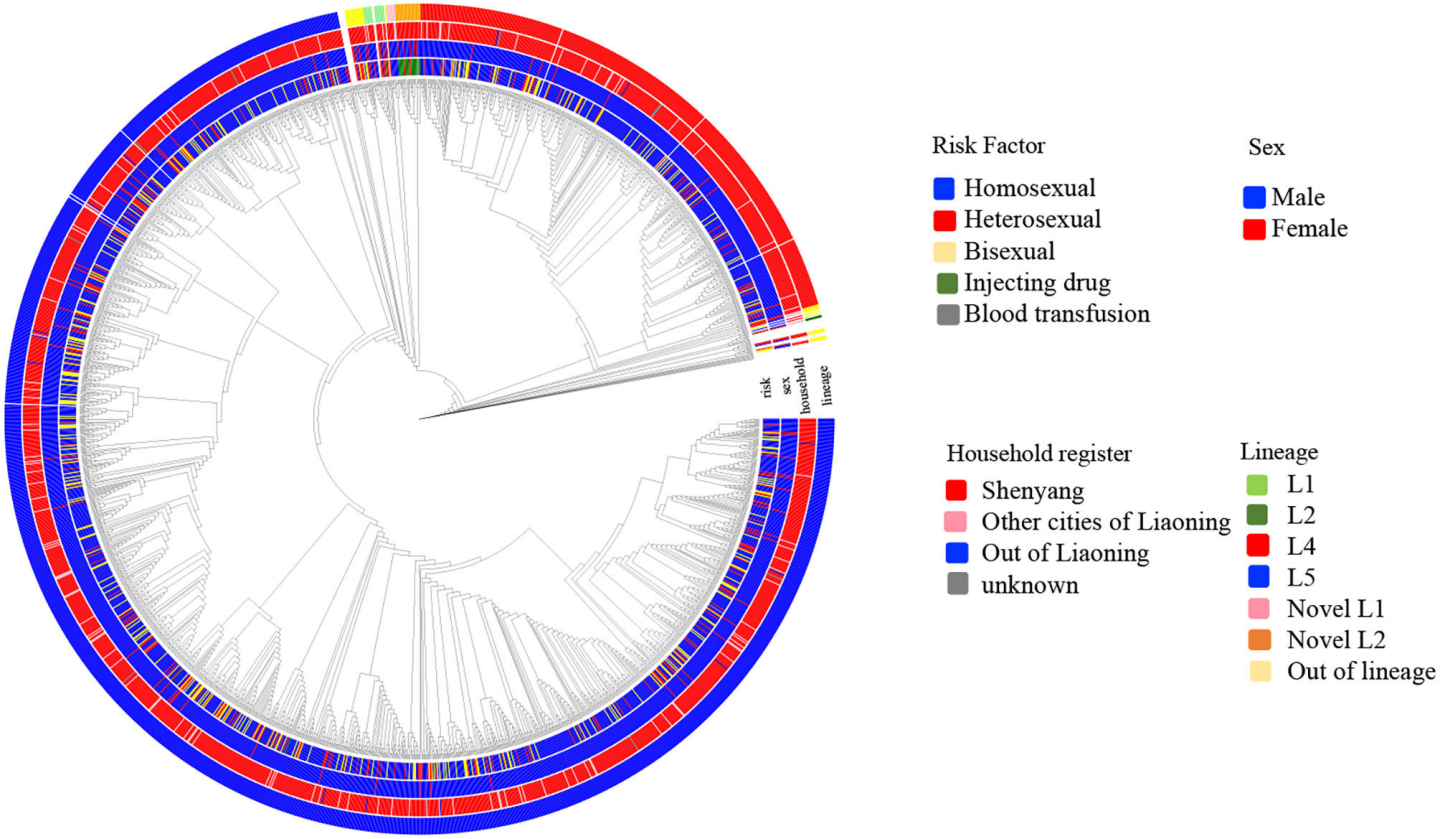
The phylogenetic reconstruction of HIV-1 CRF01_AE in Shenyang during 2002–2017. The maximum likelihood tree was constructed using 2014 HIV-1 CRF01_AE pol sequences (HXB2:2253-3314 nt) sampled in Shenyang under GTR+I+G4 model with 1000 replicates in IQ-tree. The tree is rooted with three subtype A sequences as outgroup and the reference sequences (ref.5) were used to define CRF01_AE. Six lineages were identified, of which four were reported previously. Four colored circles represent risk factor, sex, household register and lineage from inner to outer.

Six independent lineages, including 1,988 CRF01_AE sequences, were detected (Figure 1), of which four lineages have been reported previously (7). Two novel lineages were identified as circulating among homosexuals and PWID, separately. Of four previously reported lineages, two lineages (L4 and L5) were predominant, accounting for 96.4% (1,942/2,014) of cases, and were especially prevalent among homosexuals. Obviously, the proportion of L5 was much higher than that of L4 (75.3 vs. 21.2%). Compared with individuals with L4, individuals bearing L5 were older (*p* < 0.001; Table 1). During 2002–2007, both lineages were detected, but the proportion of L5 was higher than that of L4 (*p* < 0.006; Table 1).

### The Spatiotemporal History of Two Major CRF01_AE Lineages in Shenyang

Because more sequences were obtained from the homosexual and bisexual populations between 2008 and 2017, we down-sampled the number of sequences from high-density sampled homosexuals and bisexuals to ensure that the numbers of sequences associated with these two groups were equal to that associated with heterosexuals, resulting in the generation of Dataset B (Supplementary Table S1). The final sub-dataset contained 130 sequences for L4 and 576 sequences for L5. The down-sampling procedure was performed to avoid the potential for oversampling bias among MSM, including homosexuals and bisexuals, when assessing the epidemic history of each risk group and the migration events across risk groups.

The time-scaled phylogenetic trees of two lineages were reconstructed using Dataset B. The origination events linked to African and Thailand were consistent across the different risk groups for both L4 and L5, which were identified as occurring in the late 1970s and middle 1980s (Table 2). The tMRCA for the four sexual risk groups associated with L4 and for heterosexuals associated with L5 were the early 2000s in Shenyang, with evolutionary rates ranging from 2.53–2.84 ×10^−3^ substitutions/site/year. However, the tMRCA of homosexuals and bisexuals of Shenyang with L5 were traced to 1993, much earlier than another two groups of L5 and all four groups of L4, and these groups of L5 were associated with faster evolutionary rates (Table 2). The homosexuals in Shenyang had the highest root state posterior probabilities (66.56% in L4 and 93.31% in L5) (Supplementary Figure S1).

**Table 2:** Time-scaled phylogenetic analysis of two lineages among different risk groups in Shenyang.

	**Lineage 4**				**Lineage 5**			
	**Africa (95% HPD)**	**Thailand (95% HPD)**	**Shenyang (95% HPD)**	**Rate × 10^**−3**^ subs/site/year (95% HPD)**	**Africa (95% HPD)**	**Thailand (95% HPD)**	**Shenyang (95% HPD)**	**Rate × 10^**−3**^ subs/site/year (95% HPD)**
Homosexual	1,979.78	1,986.39	2,002.24	2.75	1,978.01	1,985.32	1,993.9	3.68
	(1,974.10–1,984.23)	(1,984.05–1,988.76)	(1,999.51–2,004.55)	(2.24–3.2)	(1,972.71–1,982.17)	(1,982.97–1,987/37)	(1,986.97–2,002.34)	(3.21–4.18)
Bisexual	1,979.02	19,85.89	2,001.87	2.56	1,977.97	1,985.47	1,993.29	3.26
	(1,973.68–1,983.54)	(1,983.34–1,988.16)	(1,999.20–2,004.01)	(2.05–3.14)	(1,972.81–1,982.00)	(1,983.17–1,987.85)	(1,987.29–2,002.74)	(2.78–3.72)
HET-male	1,979.65	1,986.27	2,001.97	2.61	1,976.49	1,984.42	2,001.04	2.84
	(1,973.46–1,984.46)	(1,983.72–1,988.72)	(1,999.16–2,004.58)	(2.02–3.29)	(1,970.92–1,981.24)	(1,981.63–1986.71)	(1,999.52–2002.38)	(2.40–3.27)
HET-female	1,979.91	1,986.38	2,003.06	2.53	1,977.72	1,985.09	2,002.1	2.28
	(1,972.83–1,986.63)	(1,983.05–1,989.55)	(1,997.13–2,008.09)	(1.69–3.66)	(1,972.05–1,982.16)	(1,982.49–1,987.36)	(2,000.20–2,003.66)	(1.86–2.75)

**Figure 2:**
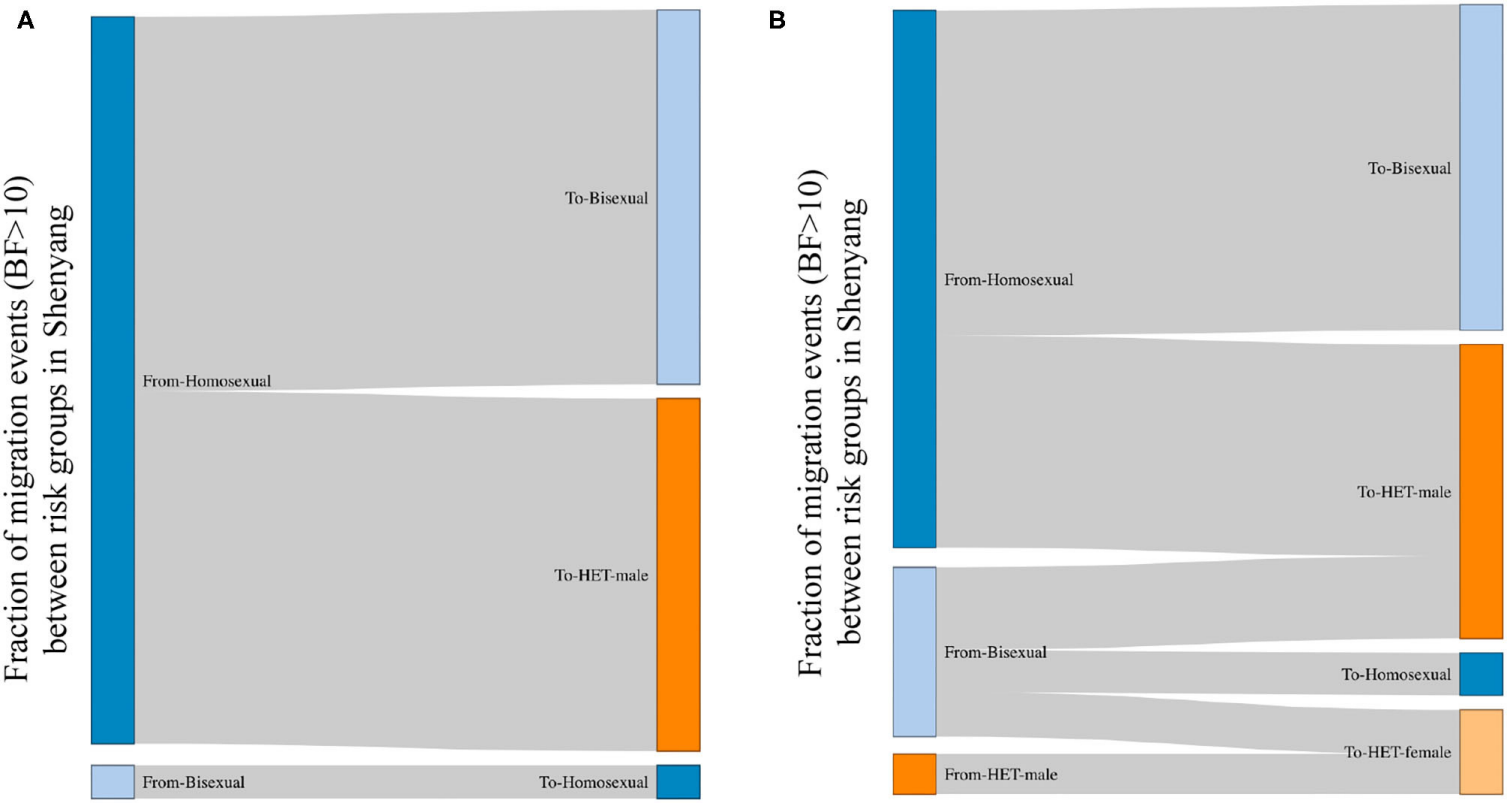
The viral migration events between risk groups in Shenyang, based on a Bayes Factor >10. BF>10 is a strong support for observed migration events; therefore, the migration events with BF >10 are shown between risk groups. The left and right sides represent the source and recipient of migration events, respectively. The fractions for each side represent the proportions of migration events from the sources toward the recipients. **(A,B)** represent the viral movements of L4 and L5, respectively, among risk groups in Shenyang.

The viral spread across risk groups could be assessed when the risk factors were used as the discrete traits. All links between sexual risk groups that were strongly supported by BF > 10 (21) are shown in Figure 2. For both L4 and L5, men were the only sources, most of whom were homosexuals (96% in L4 and 72% in L5). Compared with L4, in L5, the bisexuals and male heterosexuals were donors in almost 30% of cases. The migration events from homosexuals toward male heterosexuals accounted for almost half of all migrations for both lineages. Among recipients, the top two groups were bisexuals (49% in L4 and 44% in L5) and male heterosexuals (46% in L4 and 39% in L5), followed by female heterosexuals (0% in L4 and 11% in L5) and homosexuals (4% in L4 and 6% in L5).

### The Transmission Dynamics of Two Major CRF01_AE Lineages in Shenyang

**Figure 3:**
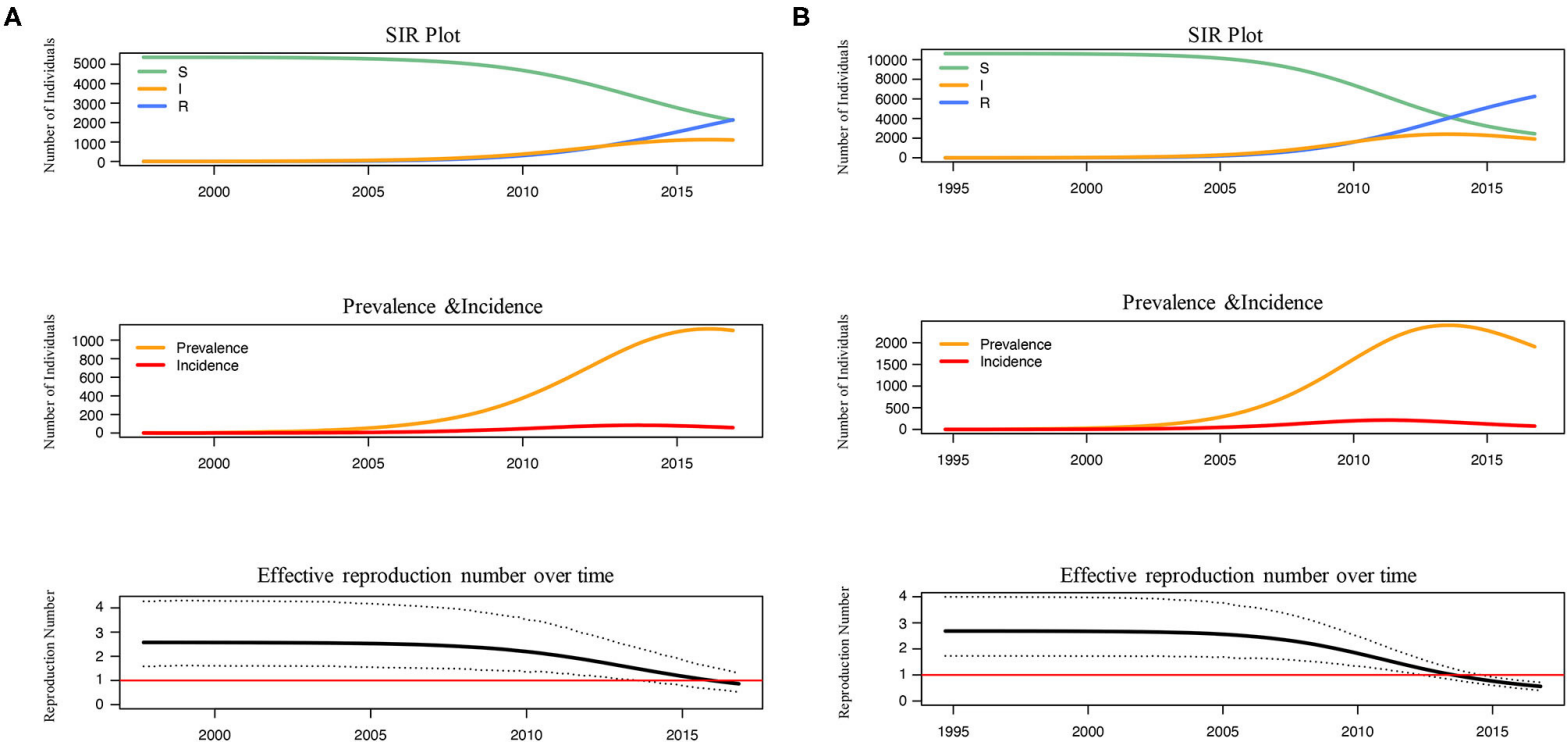
The reconstructed SIR trajectory, incidence and effective reproduction number over time of L4 **(A)** and L5 **(B)**. The transmission dynamics of L4 and L5 in Shenyang were reconstructed using BDSIR model in BEAST v.2.6.3.The changes of susceptible-infected-recovered individuals, prevalence, incidence and effective reproduction number were plotted.

The number of susceptible-infected-recovered individuals, the prevalence, the incidence and the effective reproduction number over time were estimated under BDSIR model (Figure 3). R_0_ of L4 and L5 were similar and both started to decline between 2005–2010, while the R_e_ of L5 dropped to 1 faster before 2015. Although the number of the initial susceptible individuals of both lineages was different, they had reduced since 2005. From the curve of prevalence, L4 just reached the epidemic peak and L5 had passed the peak.

## Discussion

Liaoning is among the provinces where HIV-1 positive MSM were initially reported in China, with a middle-level prevalence of HIV-1 among MSM (22). Our team has long focused on HIV-1 status of the population in Shenyang and built a local HIV-1 drug-resistance database since the early 2000s, including tens of thousands of HIV-1 *pol* sequences, accompanied by demographic and clinical details. We found that there were two CRF01_AE lineages accounting for 96.5% of all CRF01_AE infected individuals in Shenyang, but the distributions of the two lineages were significantly uneven (21 vs. 75%) (Table 1). National molecular epidemiological studies have reported that these two lineages primarily circulated among MSM, with unbalanced proportions observed in different regions (5, 7). The proportion of L5 in Northeast China was much higher than those in other regions of China, where the situation of L4 was on the contrary (7, 8, 12). Human mobility data showed that although totally there were a net out-flow of population from Northeastern provinces, mainly toward Beijing (33.3%), Guangdong (13.3%), Shanghai (11.3%) and Tianjin (10.3%), in Northeast China, Liaoning was the major destination of out-migrants from other two provinces (Jilin and Heilongjiang) (23, 24). Therefore, we suggest that HIV-1 transmission patterns in Liaoning has influenced subtype structures of its neighboring provinces to a large extent, due to the flow-in and flow-out of labor workers regionally.

Molecular clock analyses revealed that the dominant lineage (L5) had been introduced into homosexuals and bisexuals of Shenyang in 1993 (Table 1), which coincided with a free travel policy to Thailand that was allowed by Chinese government in 1990 (5). The tMRCAs of all sexual risk groups in L4 and heterosexual group in L5 were during the early 2000s (Table 1), which were later than the previous national studies, in which the origins of the two lineages were traced to the late 1990s (5, 6, 8). Moreover, compared with L4, more elders and more early sampled sequences were observed in L5 (Table 1). Additionally, the viral migration analyses found that MSM (homosexuals and bisexuals) were the primary sources for both lineages (Figure 2), indicating that the strains of these two lineages were first introduced into MSM of Shenyang, despite the divergent origin time. These results prove that for L4, Shenyang might only serve as a sink throughout the viral national transmission history, whereas for L5, MSM of Shenyang might be the recipients of the earliest viral introduction from abroad to China, which likely occurred in the early 1990s, and then served as ancestral source for viral spread toward neighboring provinces and across China, which started in the early 2000s.

Although L5 was observed among MSM in Shenyang in the early 1990s, the transmission toward heterosexuals did not appear to occur until 2001. Moreover, to the national scale, both CRF01_AE lineages begun to expand in the early 2000s, implying that the social tolerance to MSM was insufficient before 2000, which might have restricted MSM activities, resulting in low levels of HIV transmission among MSM and across risk population during the 1990s. Otherwise, the frequent transmissions from homosexuals to male heterosexuals (Figure 2) uncovered that some males who are self-reported heterosexuals might hide their true sexual orientation and also engage in sexual contact with men, resulting this population can be easily ignored when HIV/AIDS routine management is targeted toward MSM. Furthermore, the observed migration events from bisexuals and male heterosexuals to female heterosexuals revealed that the virus has entered to circulation among the general populations (Figure 2).

Finally, from the transmission dynamic history, the reductions of the number of susceptible individuals and the R_e_ for both lineages were observed after 2005 (Figure 3), which coincided to the initiation of antiretroviral therapy (ART) in 2005 and the establishment of a high-risk MSM cohort since 2008 in Shenyang. The R_e_ of both lineages have fell <1, but the decline of R_e_ within L5, the more ancient and common lineage, occurred earlier. These results proved that the ART and behavioral interventions were effective to prevent the transmission of CRF01_AE among MSM in Shenyang, especially for controlling the native-born strains.

A major limitation of phylogeographic and phylodynamic inferences is the sampling bias, such as the sequence number of different sexual risk groups as observed in this study (Supplementary Table S1). We randomly down-sampled the raw datasets from homosexual and bisexual risk groups to avoid the over-representation of sequences due to the high risk MSM cohort built since 2008 and to ensure an unbiased reconstruction (Supplementary Table S1). Additionally, for the BDSIR model, a key assumption is that keeping the population size is constant, and the growing population in Shenyang may bias the phylodynamic estimates of under the BDSIR model. The influence of the population size were equal on two lineages, therefore the observed differences of epidemic dynamics of them were still believable. Finally, with the update of treatment policy and the advanced concerns to key risk populations, the constant set of non-infectious rate in BDSIR model may under-estimate the effectiveness of interventions.

In conclusion, we reveal that Shenyang served as the ancestral source of lineage 5 in the regional and even national spread, while only a sink of lineage 4, and the sustained attention to MSM in Shenyang are still essential despite HIV-1 CRF01_AE epidemic has been controlled under cART and behavioral interventions. The results suggest that viral founder effect and the human mobility drove by economic factors may shape the viral transmission modes regionally and nationally, contributing to identify source and hotspots of HIV spread for targeting interventions.

## Data Availability Statement

The datasets presented in this study can be found in online repositories. The names of the repository/repositories and accession number(s) can be found in the article/Supplementary Material.

## Ethics Statement

The studies involving human participants were reviewed and approved by The Medical Ethics Committee of the First Hospital of China Medical University. The patients/participants provided their written informed consent to participate in this study.

## Author Contributions

HS and XH designed the study. ZC, JX, and HD collected the blood samples and demographic information. BZ and LW amplified viral sequences. MA analyzed the data and wrote the primary draft. XH revised the manuscript. The final version of this manuscript was approved by all authors. All authors contributed to the article and approved the submitted version.

## Funding

The work was supported by the Mega-projects of National Science Research for the 13th Five-Year Plan [Grant Numbers: 2017ZX10201101, 2018ZX10721102-006-003], the National Natural Science Foundation of China [Grant Number: 81871637], and CAMS Innovation Fund for Medical Sciences [Grant Number: 2019-I2M-5-027].

## Conflict of Interest

The authors declare that the research was conducted in the absence of any commercial or financial relationships that could be construed as a potential conflict of interest.

## Publisher's Note

All claims expressed in this article are solely those of the authors and do not necessarily represent those of their affiliated organizations, or those of the publisher, the editors and the reviewers. Any product that may be evaluated in this article, or claim that may be made by its manufacturer, is not guaranteed or endorsed by the publisher.
