# Supplementary material for: The Viral Founder Effect and Economic-Driven Human Mobility Shaped the Distinct Epidemic Pattern of HIV-1 CRF01_AE in Northeast China
Source: Front Med (Lausanne). 2021 Dec 3;8:769535. doi: 10.3389/fmed.2021.769535 (PMC8678122; doi:10.3389/fmed.2021.769535)
Supplement: Supplementary file 1 [file Data_Sheet_1.docx]

**Supplementary material:**

Table S1 The numbers of sequences before and after down-sampling for two lineages in Shenyang. The number of sequences after down-sampling were highlighted in bold.

|  |  | < 2008 |  | >= 2008 | |  | total |
| --- | --- | --- | --- | --- | --- | --- | --- |
|  |  | —— |  | pre | post |  |  |
| Lineage 4 | Homosexual | 2 |  | 324 | **42** |  | **130** |
|  | Bisexual | 1 |  | 56 | **42** |  |  |
|  | HET | 0 |  | 42 | 42 |  |  |
|  | IDU | 0 |  | 0 | 0 |  |  |
|  | Blood | 0 |  | 1 | 1 |  |  |
| Lineage 5 | Homosexual | 20 |  | 1057 | **179** |  | **576** |
|  | Bisexual | 4 |  | 241 | **179** |  |  |
|  | HET | 10 |  | 179 | 179 |  |  |
|  | IDU | 1 |  | 1 | 1 |  |  |
|  | Blood | 0 |  | 3 | 3 |  |  |

Figure S1 The root state posterior probability among risk groups estimated in MCC tree for Lineage 4 and Lineage 5 in Shenyang

The accession numbers of sequences used in this study

FJ531381 FJ531383 FJ531391 FJ531397 FJ531408 FJ531409 FJ531416 FJ531417 FJ531420 FJ531422 FJ531424 FJ531431 FJ531435 FJ531436 FJ531448 FJ531455 FJ531462 FJ531470 KC888252 KC888289 KC888289 KC888289 KC888316 KC888317 KC888325 KC888327 KC888329 MT368089 MT368217 MT368218 MT368219 MT368220 MT368221 MT368222 MT368223 MT368224 MT368225 MT368226 MT368227 MT368228 MT368229 MT368230 MT368231 MT368232 MT368233 MT368234 MT368235 MT368236 MT368237 MT368238 MT368239 MT368240 MT368241 MT368242 MT368243 MT368244 MT368245 MT368246 MT368247 MT368248 MT368249 MT368250 MT368252 MT368253 MT368254 MT368255 MT368256 MT368257 MT368258 MT368259 MT368260 MT368261 MT368262 MT368263 MT368264 MT368265 MT368267 MT368268 MT368269 MT368270 MT368271 MT368272 MT368273 MT368274 MT368275 MT368276 MT368277 MT368278 MT368279 MT368280 MT368281 MT368282 MT368283 MT368284 MT368286 MT368287 MT368288 MT368289 MT368290 MT368291 MT368292 MT368293 MT368295 MT368296 MT368297 MT368300 MT368301 MT368302 MT368304 MT368305 MT368306 MT368307 MT368309 MT368310 MT368311 MT368312 MT368313 MT368314 MT368315 MT368316 MT368317 MT368318 MT368319 MT368320 MT368321 MT368322 MT368323 MT368324 MT368325 MT368326 MT368327 MT368328 MT368329 MT368330 MT368331 MT368332 MT368333 MT368334 MT368335 MT368336 MT368337 MT368338 MT368339 MT368341 MT368342 MT368343 MT368344 MT368345 MT368346 MT368347 MT368348 MT368349 MT368350 MT368351 MT368352 MT368353 MT368354 MT368355 MT368356 MT368357 MT368358 MT368359 MT368360 MT368361 MT368362 MT368363 MT368364 MT368365 MT368366 MT368367 MT368368 MT368369 MT368370 MT368371 MT368372 MT368373 MT368374 MT368375 MT368376 MT368377 MT368378 MT368379 MT368380 MT368381 MT368382 MT368383 MT368384 MT368385 MT368386 MT368387 MT368388 MT368389 MT368390 MT368391 MT368392 MT368393 MT368394 MT368395 MT368396 MT368397 MT368398 MT368399 MT368400 MT368401 MT368402 MT368403 MT368404 MT368405 MT368406 MT368407 MT368408 MT368409 MT368410 MT368411 MT368412 MT368413 MT368414 MT368415 MT368416 MT368417 MT368418 MT368419 MT368420 MT368421 MT368422 MT368423 MT368424 MT368425 MT368426 MT368427 MT368428 MT368429 MT368430 MT368431 MT368432 MT368433 MT368434 MT368435 MT368436 MT368437 MT368439 MT368440 MT368441 MT368442 MT368443 MT368444 MT368445 MT368446 MT368447 MT368448 MT368449 MT368450 MT368451 MT368452 MT368453 MT368454 MT368455 MT368456 MT368457 MT368458 MT368459 MT368460 MT368461 MT368462 MT368463 MT368464 MT368465 MT368466 MT368467 MT368468 MT368469 MT368470 MT368471 MT368472 MT368473 MT368474 MT368475 MT368476 MT368477 MT368478 MT368479 MT368480 MT368481 MT368482 MT368483 MT368484 MT368485 MT368486 MT368487 MT368488 MT368489 MT368490 MT368491 MT368492 MT368493 MT368494 MT368495 MT368496 MT368497 MT368498 MT368499 MT368500 MT368501 MT368502 MT368503 MT368504 MT368505 MT368506 MT368507 MT368508 MT368509 MT368510 MT368511 MT368512 MT368513 MT368514 MT368515 MT368516 MT368517 MT368518 MT368519 MT368520 MT368521 MT368522 MT368523 MT368524 MT368525 MT368526 MT368527 MT368528 MT368529 MT368530 MT368531 MT368532 MT368533 MT368534 MT368535 MT368537 MT368538 MT368539 MT368540 MT368541 MT368542 MT368543 MT368544 MT368545 MT368546 MT368548 MT368549 MT368551 MT368552 MT368553 MT368554 MT368555 MT368556 MT368557 MT368558 MT368559 MT368560 MT368561 MT368562 MT368563 MT368564 MT368565 MT368566 MT368567 MT368568 MT368569 MT368570 MT368571 MT368573 MT368574 MT368576 MT368577 MT368578 MT368579 MT368580 MT368581 MT368582 MT368583 MT368584 MT368585 MT368586 MT368587 MT368588 MT368589 MT368590 MT368591 MT368592 MT368593 MT368594 MT368595 MT368596 MT368597 MT368598 MT368599 MT368600 MT368601 MT368602 MT368603 MT368604 MT368605 MT368606 MT368607 MT368608 MT368609 MT368611 MT368612 MT368613 MT368614 MT368615 MT368616 MT368617 MT368618 MT368619 MT368620 MT368621 MT368622 MT368623 MT368624 MT368625 MT368626 MT368627 MT368628 MT368629 MT368630 MT368632 MT368633 MT368634 MT368635 MT368636 MT368637 MT368638 MT368639 MT368640 MT368641 MT368642 MT368643 MT368644 MT368645 MT368646 MT368647 MT368648 MT368649 MT368650 MT368651 MT368652 MT368653 MT368655 MT368656 MT368657 MT368658 MT368659 MT368660 MT368661 MT368662 MT368663 MT368664 MT368665 MT368666 MT368667 MT368668 MT368669 MT368670 MT368671 MT368672 MT368673 MT368674 MT368675 MT368676 MT368677 MT368678 MT368679 MT368680 MT368681 MT368682 MT368683 MT368684 MT368685 MT368686 MT368687 MT368688 MT368689 MT368690 MT368691 MT368692 MT368693 MT368694 MT368695 MT368696 MT368697 MT368698 MT368699 MT368700 MT368701 MT368702 MT368703 MT368704 MT368706 MT368707 MT368708 MT368710 MT368711 MT368712 MT368713 MT368714 MT368715 MT368716 MT368717 MT368718 MT368719 MT368720 MT368721 MT368722 MT368723 MT368724 MT368725 MT368726 MT368727 MT368728 MT368729 MT368730 MT368731 MT368732 MT368733 MT368734 MT368735 MT368736 MT368737 MT368738 MT368739 MT368740 MT368741 MT368742 MT368743 MT368744 MT368745 MT368746 MT368747 MT368748 MT368749 MT368750 MT368751 MT368752 MT368753 MT368754 MT368755 MT368756 MT368757 MT368758 MT368759 MT368760 MT368761 MT368762 MT368763 MT368764 MT368765 MT368766 MT368767 MT368768 MT368769 MT368770 MT368771 MT368772 MT368773 MT368774 MT368775 MT368776 MT368777 MT368778 MT368779 MT368780 MT368781 MT368782 MT368783 MT368784 MT368785 MT368786 MT368787 MT368788 MT368789 MT368790 MT368791 MT368792 MT368793 MT368794 MT368795 MT368796 MT368797 MT368798 MT368799 MT368800 MT368801 MT368802 MT368803 MT368804 MT368805 MT368806 MT368807 MT368808 MT368809 MT368810 MT368811 MT368812 MT368813 MT368814 MT368815 MT368816 MT368817 MT368818 MT368819 MT368820 MT368821 MT368822 MT368824 MT368825 MT368826 MT368827 MT368828 MT368829 MT368830 MT368831 MT368832 MT368833 MT368834 MT368835 MT368836 MT368837 MT368838 MT368839 MT368840 MT368841 MT368842 MT368843 MT368844 MT368845 MT368846 MT368847 MT368848 MT368849 MT368850 MT368851 MT368852 MT368853 MT368854 MT368855 MT368856 MT368857 MT368858 MT368859 MT368860 MT368861 MT368862 MT368863 MT368864 MT368865 MT368866 MT368867 MT368868 MT368869 MT368870 MT368871 MT368872 MT368873 MT368874 MT368875 MT368876 MT368877 MT368878 MT368879 MT368880 MT368881 MT368882 MT368883 MT368884 MT368885 MT368886 MT368887 MT368888 MT368889 MT368890 MT368891 MT368892 MT368893 MT368894 MT368895 MT368896 MT368897 MT368898 MT368899 MT368900 MT368901 MT368902 MT368903 MT368904 MT368905 MT368906 MT368907 MT368908 MT368909 MT368910 MT368911 MT368912 MT368913 MT368914 MT368915 MT368916 MT368917 MT368918 MT368919 MT368920 MT368921 MT368922 MT368923 MT368924 MT368925 MT368926 MT368927 MT368928 MT368929 MT368930 MT368931 MT368932 MT368933 MT368934 MT368935 MT368936 MT368937 MT368938 MT368939 MT368940 MT368941 MT368942 MT368943 MT368944 MT368945 MT368946 MT368947 MT368948 MT368949 MT368950 MT368951 MT368952 MT368953 MT368954 MT368955 MT368956 MT368957 MT368958 MT368959 MT368960 MT368961 MT368962 MT368963 MT368964 MT368965 MT368966 MT368967 MT368968 MT368969 MT368970 MT368971 MT368972 MT368973 MT368974 MT368975 MT368976 MT368977 MT368978 MT368979 MT368980 MT368981 MT368982 MT368983 MT368984 MT368985 MT368986 MT368987 MT368988 MT368989 MT368990 MT368992 MT368993 MT368994 MT368995 MT368996 MT368997 MT368998 MT368999 MT369000 MT369001 MT369002 MT369003 MT369004 MT369005 MT369006 MT369007 MT369008 MT369009 MT369010 MT369011 MT369012 MT369013 MT369014 MT369015 MT369016 MT369017 MT369018 MT369019 MT369020 MT369021 MT369022 MT369023 MT369024 MT369025 MT369026 MT369027 MT369028 MT369029 MT369030 MT369031 MT369032 MT369033 MT369034 MT369035 MT369036 MT369037 MT369038 MT369039 MT369040 MT369041 MT369042 MT369043 MT369044 MT369045 MT369046 MT369047 MT369048 MT369049 MT369050 MT369051 MT369052 MT369053 MT369054 MT369055 MT369056 MT369057 MT369058 MT369059 MT369060 MT369061 MT369062 MT369063 MT369064 MT369065 MT369066 MT369067 MT369068 MT369069 MT369070 MT369071 MT369072 MT369073 MT369074 MT369075 MT369076 MT369077 MT369078 MT369079 MT369080 MT369081 MT369082 MT369083 MT369084 MT369085 MT369086 MT369087 MT369088 MT369089 MT369090 MT369091 MT369092 MT369093 MT369094 MT369095 MT369096 MT369097 MT369098 MT369099 MT369100 MT369101 MT369102 MT369103 MT369104 MT369105 MT369106 MT369107 MT369108 MT369109 MT369110 MT369111 MT369112 MT369113 MT369114 MT369115 MT369116 MT369117 MT369118 MT369119 MT369120 MT369121 MT369122 MT369123 MT369124 MT369125 MT369126 MT369127 MT369128 MT369129 MT369130 MT369131 MT369132 MT369133 MT369134 MT369135 MT369136 MT369137 MT369138 MT369139 MT369140 MT369141 MT369142 MT369143 MT369144 MT369145 MT369146 MT369148 MT369149 MT369150 MT369151 MT369152 MT369153 MT369154 MT369155 MT369156 MT369157 MT369158 MT369159 MT369160 MT369161 MT369163 MT369164 MT369165 MT369166 MT369167 MT369168 MT369169 MT369170 MT369171 MT369172 MT369173 MT369174 MT369175 MT369176 MT369177 MT369178 MT369179 MT369180 MT369181 MT369182 MT369183 MT369184 MT369185 MT369186 MT369187 MT369188 MT369189 MT369190 MT369191 MT369192 MT369193 MT369194 MT369195 MT369196 MT369197 MT369198 MT369199 MT369200 MT369201 MT369202 MT369203 MT369204 MT369205 MT369206 MT369207 MT369208 MT369209 MT369210 MT369211 MT369212 MT369213 MT369214 MT369215 MT369216 MT369217 MT369218 MT369219 MT369220 MT369221 MT369222 MT369223 MT369224 MT369225 MT369226 MT369227 MT369228 MT369229 MT369230 MT369231 MT369232 MT369233 MT369234 MT369235 MT369236 MT369237 MT369238 MT369239 MT369240 MT369241 MT369242 MT369243 MT369244 MT369245 MT369246 MT369247 MT369248 MT369249 MT369250 MT369251 MT369252 MT369253 MT369254 MT369255 MT369256 MT369257 MT369258 MT369259 MT369260 MT369261 MT369262 MT369263 MT369264 MT369265 MT369266 MT369267 MT369268 MT369269 MT369270 MT369271 MT369272 MT369273 MT369274 MT369275 MT369276 MT369277 MT369278 MT369279 MT369280 MT369281 MT369282 MT369283 MT369284 MT369285 MT369286 MT369287 MT369288 MT369289 MT369290 MT369291 MT369292 MT369293 MT369294 MT369295 MT369296 MT369297 MT369298 MT369299 MT369300 MT369301 MT369302 MT369303 MT369304 MT369305 MT369306 MT369307 MT369308 MT369309 MT369310 MT369311 MT369312 MT369313 MT369314 MT369315 MT369316 MT369317 MT369318 MT369319 MT369320 MT369321 MT369322 MT369323 MT369324 MT369325 MT369326 MT369327 MT369328 MT369329 MT369330 MT369331 MT369332 MT369333 MT369334 MT369335 MT369336 MT369337 MT369338 MT369339 MT369340 MT369341 MT369342 MT369343 MT369344 MT369345 MT369346 MT369347 MT369348 MT369349 MT369350 MT369351 MT369352 MT369353 MT369354 MT369355 MT369356 MT369357 MT369358 MT369359 MT369360 MT369361 MT369362 MT369363 MT369365 MT369366 MT369367 MT369368 MT369369 MT369370 MT369371 MT369372 MT369373 MT369374 MT369375 MT369376 MT369377 MT369378 MT369379 MT369380 MT369381 MT369382 MT369383 MT369384 MT369385 MT369386 MT369387 MT369388 MT369389 MT369390 MT369391 MT369392 MT369393 MT369394 MT369395 MT369396 MT369397 MT369398 MT369399 MT369400 MT369401 MT369402 MT369403 MT369404 MT369405 MT369406 MT369407 MT369408 MT369409 MT369410 MT369411 MT369412 MT369413 MT369414 MT369415 MT369416 MT369417 MT369418 MT369419 MT369420 MT369421 MT369422 MT369424 MT369425 MT369426 MT369427 MT369428 MT369429 MT369430 MT369431 MT369432 MT369433 MT369434 MT369435 MT369436 MT369437 MT369438 MT369439 MT369440 MT369441 MT369442 MT369443 MT369444 MT369445 MT369446 MT369447 MT369448 MT369449 MT369450 MT369451 MT369452 MT369453 MT369454 MT369455 MT369456 MT369457 MT369458 MT369459 MT369460 MT369461 MT369462 MT369463 MT369464 MT369465 MT369466 MT369467 MT369468 MT369469 MT369470 MT369471 MT369472 MT369473 MT369474 MT369475 MT369476 MT369477 MT369478 MT369479 MT369480 MT369481 MT369482 MT369483 MT369484 MT369485 MT369486 MT369487 MT369488 MT369489 MT369490 MT369491 MT369492 MT369493 MT369494 MT369495 MT369496 MT369497 MT369498 MT369499 MT369500 MT369501 MT369503 MT369504 MT369505 MT369506 MT369507 MT369508 MT369509 MT369510 MT369511 MT369512 MT369513 MT369514 MT369515 MT369516 MT369517 MT369518 MT369519 MT369520 MT369521 MT369522 MT369523 MT369524 MT369525 MT369526 MT369527 MT369528 MT369530 MT369531 MT369532 MT369533 MT369534 MT369535 MT369536 MT369537 MT369538 MT369539 MT369540 MT369541 MT369542 MT369544 MT369545 MT369546 MT369547 MT369548 MT369549 MT369550 MT369551 MT369552 MT369553 MT369554 MT369555 MT369556 MT369557 MT369558 MT369559 MT369560 MT369561 MT369562 MT369563 MT369564 MT369565 MT369566 MT369567 MT369568 MT369569 MT369570 MT369571 MT369572 MT369573 MT369575 MT369576 MT369577 MT369578 MT369579 MT369580 MT369581 MT369582 MT369583 MT369585 MT369586 MT369587 MT369588 MT369589 MT369590 MT369591 MT369592 MT369593 MT369594 MT369595 MT369598 MT369599 MT369600 MT369601 MT369602 MT369603 MT369604 MT369605 MT369606 MT369607 MT369608 MT369609 MT369610 MT369611 MT369612 MT369613 MT369614 MT369615 MT369616 MT369617 MT369618 MT369619 MT369620 MT369621 MT369622 MT369623 MT369624 MT369625 MT369627 MT369628 MT369629 MT369630 MT369631 MT369632 MT369633 MT369634 MT369635 MT369636 MT369637 MT369638 MT369639 MT369640 MT369641 MT369643 MT369644 MT369645 MT369646 MT369647 MT369648 MT369649 MT369650 MT369651 MT369652 MT369653 MT369654 MT369655 MT369656 MT369657 MT369658 MT369659 MT369660 MT369661 MT369662 MT369663 MT369665 MT369666 MT369667 MT369668 MT369669 MT369670 MT369671 MT369672 MT369673 MT369674 MT369675 MT369676 MT369677 MT369678 MT369679 MT369680 MT369681 MT369682 MT369683 MT369684 MT369685 MT369686 MT369687 MT369688 MT369689 MT369690 MT369691 MT369692 MT369693 MT369694 MT369695 MT369696 MT369697 MT369698 MT369699 MT369700 MT369701 MT369702 MT369703 MT369704 MT369705 MT369706 MT369707 MT369708 MT369709 MT369710 MT369711 MT369712 MT369713 MT369714 MT369715 MT369716 MT369717 MT369718 MT369719 MT369720 MT369721 MT369722 MT369723 MT369724 MT369725 MT369726 MT369727 MT369728 MT369729 MT369730 MT369731 MT369732 MT369733 MT369734 MT369735 MT369736 MT369737 MT369738 MT369739 MT369740 MT369741 MT369742 MT369743 MT369744 MT369745 MT369746 MT369747 MT369748 MT369749 MT369750 MT369751 MT369752 MT369753 MT369754 MT369755 MT369756 MT369757 MT369758 MT369759 MT369760 MT369761 MT369762 MT369763 MT369764 MT369765 MT369766 MT369767 MT369768 MT369769 MT369770 MT369772 MT369773 MT369774 MT369775 MT369776 MT369777 MT369778 MT369779 MT369780 MT369781 MT369782 MT369783 MT369784 MT369785 MT369786 MT369787 MT369788 MT369789 MT369790 MT369791 MT369792 MT369793 MT369794 MT369795 MT369796 MT369797 MT369798 MT369799 MT369800 MT369801 MT369802 MT369803 MT369804 MT369805 MT369806 MT369807 MT369808 MT369809 MT369814 MT369816 MT369817 MT369818 MT369820 MT369821 MT369822 MT369823 MT369824 MT369826 MT369827 MT369828 MT369829 MT369830 MT369831 MT369832 MT369833 MT369834 MT369835 MT369836 MT369837 MT369839 MT369840 MT369841 MT369842 MT369844 MT369845 MT369846 MT369848 MT369849 MT369850 MT369851 MT369853 MT369854 MT369855 MT369856 MT369857 MT369863 MT369865 MT369867 MT369871 MT369873 MT369874 MT369875 MT369876 MT369877 MT369878 MT369879 MT369880 MT369881 MT369882 MT369883 MT369884 MT369885 MT369887 MT369888 MT369889 MT369890 MT369891 MT369892 MT369893 MT369894 MT369895 MT369896 MT369897 MT369898 MT369899 MT369900 MT369901 MT369902 MT369903 MT369904 MT369905 MT369906 MT369907 MT369908 MT369909 MT369910 MT369911 MT369912 MT369913 MT369914 MT369915 MT369916 MT369917 MT369918 MT369919 MT369920 MT369921 MT369922 MT369923 MT369924 MT369925 MT369926 MT369927
